# Oxidative Imbalance in *Candida tropicalis* Biofilms and Its Relation With Persister Cells

**DOI:** 10.3389/fmicb.2020.598834

**Published:** 2021-02-02

**Authors:** María A. da Silva, José L. Baronetti, Paulina L. Páez, María G. Paraje

**Affiliations:** ^1^Instituto Multidisciplinario de Biología Vegetal (IMBIV), Consejo Nacional de Investigaciones Científicas y Técnicas (CONICET), Córdoba, Argentina; ^2^Cátedra de Microbiología, Facultad de Ciencias Exactas, Físicas y Naturales, Universidad Nacional de Córdoba, Córdoba, Argentina; ^3^Unidad de Investigación y Desarrollo en Tecnología Farmacéutica (UNITEFA), Consejo Nacional de Investigaciones Científicas y Técnicas (CONICET), Córdoba, Argentina; ^4^Departamento de Ciencias Farmacéuticas, Facultad de Ciencias Químicas, Universidad Nacional de Córdoba, Córdoba, Argentina

**Keywords:** *Candida tropicalis*, persister cells, biofilms, oxidative stress, nitrosative stress, antifungals

## Abstract

**Background:**

Persister cells (PCs) make up a small fraction of microbial population, can survive lethal concentrations of antimicrobial agents. In recent years, *Candida tropicalis* has emerged as being a frequent fungal agent of medical devices subject to biofilm infections. However, PCs are still poorly understood.

**Objectives:**

This study aimed to investigate the relation of PCs on the redox status in *C. tropicalis* biofilms exposed to high doses of Amphotericin B (AmB), and alterations in surface topography and the architecture of biofilms.

**Methods:**

We used an experimental model of two different *C. tropicalis* biofilms exposed to AmB at supra minimum inhibitory concentration (SMIC80), and the intra- and extracellular reactive oxygen species (iROS and eROS), reactive nitrogen species (RNS) and oxidative stress response were studied. Light microscopy (LM) and confocal laser scanning microscopy (CLSM) were also used in conjunction with the image analysis software COMSTAT.

**Results:**

We demonstrated that biofilms derived from the PC fraction (B2) showed a higher capacity to respond to the stress generated upon AmB treatment, compared with biofilms obtained from planktonic cells. In B2, a lower ROS and RNS accumulation was observed in concordance with higher activation of the antioxidant systems, resulting in an oxidative imbalance of a smaller magnitude compared to B1. LM analysis revealed that the AmB treatment provoked a marked decrease of biomass, showing a loss of cellular aggrupation, with the presence of mostly yeast cells. Moreover, significant structural changes in the biofilm architecture were noted between both biofilms by CLSM—COMSTAT analysis. For B1, the quantitative parameters bio-volume, average micro-colony volume, surface to bio-volume ratio and surface coverage showed reductions upon AmB treatment, whereas increases were observed in roughness coefficient and average diffusion distance. In addition, untreated B2 was substantially smaller than B1, with less biomass and thickness values. The analysis of the above-mentioned parameters also showed changes in B2 upon AmB exposure.

**Conclusion:**

To our knowledge, this is the first study that has attempted to correlate PCs of *Candida* biofilms with alterations in the prooxidant-antioxidant balance and the architecture of the biofilms. The finding of regular and PCs with different cellular stress status may help to solve the puzzle of biofilm resistance, with redox imbalance possibly being an important factor.

## Introduction

Persister cells (PCs) are a small fraction of a microbial population and can survive lethal concentrations of antimicrobial agents, typically resulting in a biphasic killing pattern (Bigger, 1944; Keren et al., 2004; Lewis, 2010). These cells are phenotypic variants of a wild type that upon re-inoculation produce a culture with a similarly small proportion of PCs of between 0.001 and 1% of the total cell population, which are transiently refractory to killing, without having acquired resistance through genetic modification (Maisonneuve and Gerdes, 2014; Brauner et al., 2016). In addition, they have been found within biofilms and may be associated with recurrent or chronic infections (Fauvart et al., 2011; Defraine et al., 2018; Lin et al., 2018). Concerning this, PCs have been studied extensively in prokaryotic cells, and to a more limited extent in eukaryote cells, including species of the opportunistic pathogen *Candida* (LaFleur et al., 2006; LaFleur et al., 2010; Chung and Ko, 2019). Nevertheless, in contrast with bacteria, no PCs have yet been isolated from *Candida* planktonic cultures, and their presence in biofilms is disputed. Although PCs have been reported in *Candida albicans* biofilms when exposed to high concentrations of antifungals agents, other authors have been unable to find PCs in some reference and clinical strains of *Candida glabrata* and *Candida tropicalis* (Al-Dhaheri and Douglas, 2008; Denega et al., 2019).

Over the last decade, it has been reported that persistence is a highly dynamic phenotype affected by different parameters, of which antimicrobial treatments, starvation, carbon source transitions, toxic metals, and acid responses and osmotic or oxidative stresses may be able to induce persistence in regular sessile cells (Harrison et al., 2007; Lewis, 2010; Nguyen et al., 2011; Leung and Levesque, 2012; Wu et al., 2012; Amato et al., 2013; Li et al., 2015; Conlon et al., 2016; Harms et al., 2016; Amoh et al., 2017; Van den Bergh et al., 2017; Defraine et al., 2018). However, the mechanism of PC formation is not well understood, and conflicting findings have been reported concerning their presence and metabolic state (Balaban et al., 2013; Maisonneuve and Gerdes, 2014; Wuyts et al., 2018). Thus, an improved understanding of the role of fungal PCs inside biofilms would be a contribution toward providing better clinical treatment of these recalcitrant infections.

Amphotericin B (AmB), a drug known as the “gold standard” antifungal agent, with a well-known membrane-perturbing activity through interaction with ergosterol and pore formation, has been demonstrated to increase reactive oxygen species (ROS) (Mesa-Arango et al., 2014; Guirao-Abad et al., 2017). Oxidative stress is caused by the overproduction of free radicals or/and by an insufficient oxidative stress response (OSR) (Bink et al., 2011). Concerning this, a disturbance in this prooxidant/antioxidant balance in favor of the overproduction of ROS or/and reactive nitrogen species (RNS) can result in damage to the cellular components, including carbohydrates, lipids, proteins, and nucleic acids (Dixon and Stockwell, 2013). If this damage is not repaired, apoptosis and/or necrosis can occur. In addition, AmB kills fungal cells at all stages of growth, making it the optimum choice to isolate and study PCs (LaFleur et al., 2006, 2010; Al-Dhaheri and Douglas, 2008; Chung and Ko, 2019).

**FIGURE 1:**
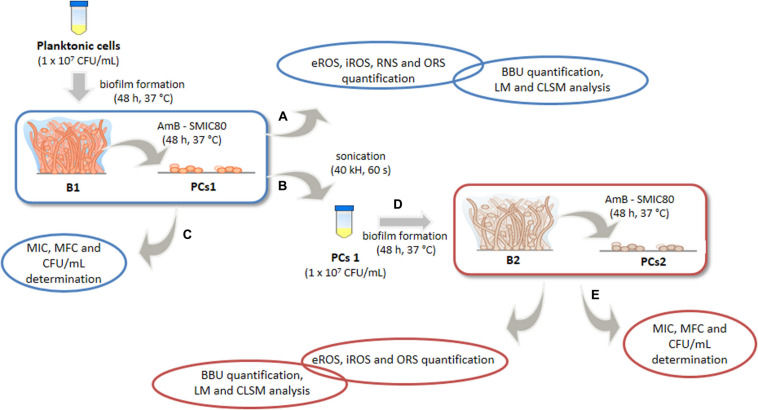
Experimental model for the study of oxidative, nitrosative stress and antioxidant stress responses (OSR) of *Candida tropicalis* biofilms formed from planktonic or persister cells (PCs). Biofilm 1 (B1) was formed from planktonic culture and exposed to Amphotericin B (AmB, 200 μg mL^–1^), and biofilm 2 (B2) was formed from the PCs1 population that survived AmB treatment in B1 and was subsequently exposed to a second AmB treatment (200 μg mL^–1^). Biofilm biomass unit (BBU) was quantified by adhesion to a 96-well microplate and with crystal violet (CV) staining, and the PC fractions were determined by colony-forming units (CFU mL^–1^). The minimum inhibitory concentration (MIC), the minimum fungicidal concentration (MFC) and the sessile minimum inhibitory concentration 80 (SMIC80) of AmB were determined. The extra and intracellular reactive oxygen species (eROS and iROS), reactive nitrogen species (RNS) and OSR from both biofilms were compared. Changes in biofilm structural parameters were analyzed by light microscope (LM) and confocal laser scanning microscopy (CLSM) using COMSTAT image-analysis software.

The present study aimed at evaluating the relation of PCs on the redox status in *C. tropicalis* biofilms exposed to high doses of AmB and their possible role in resistance to antifungal treatment. For this purpose, we used an experimental model (Figure 1) to examine the relationships among biofilms, PCs and prooxidant-antioxidant balance. We demonstrated that biofilms derived from the PC fraction (PCs1) showed a higher capacity to respond to the stress generated upon AmB treatment, compared with biofilms obtained from planktonic cells (B1). In the biofilms derived from this PC fraction (B2), a lower ROS and RNS accumulation were observed, resulting in an oxidative imbalance of a smaller magnitude. Moreover, significant surface topography differences were noted between both biofilms (B1 and B2) using confocal laser scanning microscopy (CLSM) in conjunction with the image analysis software COMSTAT. To our knowledge, this is the first study that has attempted to correlate PCs of *Candida* biofilms with alterations in the prooxidant-antioxidant balance and the architecture of the biofilms. The finding of sessile cells (regular or persister) with different cellular stress status may help to solve the puzzle of biofilm resistance, with the oxidative and nitrosative stress imbalances being possibly important factors.

## Materials and Methods

### Fungal Strains and Growth Conditions

Two *C. tropicalis* strains were tested: the reference *C. tropicalis* NCPF 3111 strain (N° 1) (National Collection of Pathogenic Fungi, Bristol, United Kingdom) and a clinical isolate (N° 2) associated with indwelling medical device infection, which were generously identified by the Microbiology Laboratory of Clinical Fabiola Queen (Córdoba, Argentina). For long-term storage, these strains were preserved in Sabouraud Dextrose Broth (SDB) (Difco, Detroit, MI, United States) with 15% glycerol at −80°C. Prior to all assays, the strains were sub-cultured from this stock onto Sabouraud Dextrose Agar (SDA—Difco, Detroit, MI, United States) at 37°C for purity and viability controls. Standardized cellular suspensions (1 × 107 cells mL^–1^) in SDB were obtained from an overnight culture at 37°C (CLSI, 2017; Marioni et al., 2017; Peralta et al., 2017).

### Antifungal Susceptibility Assay in Planktonic Cells

Minimum inhibitory concentration (MIC) and minimum fungicidal concentration (MFC) determination of AmB (Sigma-Aldrich Co., St. Louis, MO, United States) was performed according to M27-A3 guidelines of the Clinical and Laboratory Standards Institute (CLSI, 2017). The MIC was defined as the lowest concentration of the compound able to produce growth inhibition (without visible microbial growth) after 48 h of incubation, with the lowest concentration that eliminated 99.9% of the initial inoculum determined as the MFC (Marioni et al., 2016, 2017). The yeast inoculum corresponding to 0.5 on the McFarland scale and the AmB serial dilutions (0.6–64.0 μg mL^–1^) were prepared in Roswell Park Memorial Institute medium (Sigma-Aldrich Co., St. Louis, MO, United States) with 0.2% glucose and glutamine, and buffered with morpholino propanesulphonic acid (Sigma-Aldrich Co., St. Louis, MO, United States) (0.164 M) adjusted to pH 7.0 ± 0.1. Yeast suspension (100 μL) and AmB solutions were placed in the 96-well microplate (Greiner Bio-One, Frickenhausen, Germany) in triplicate and incubated 48 h at 37°C (Marioni et al., 2016, 2017). The MIC value was evaluated visually and confirmed by measuring the optical density (OD) at 490 nm. To determine the MFC value, 100 μL from wells with no visible growth were seeded onto SDA plates and subsequently incubated for 48 h at 37°C.

### Antifungal Susceptibility Assay in Biofilms

Biofilm formation was achieved through the ability of microorganisms to adhere to wells of flat-bottomed 96-well microplates, and quantified by Crystal violet (CV, Anedra Tigre, Argentina) staining at 595 nm by using a microplate reader (Infinite F50 Model, Tecan, AUS). A modification of a method previously described by O’Toole and Kolter (1998) was made by using fetal bovine serum (Greiner Bio-One, Frickenhausen, Germany) 50% (v/v) pre-treatment for 30 min at 37°C. Then, 200 μL of *C. tropicalis* standardized suspension (1 × 10^7^ cells mL^–1^) were added to each well and the plate was incubated for 90 min at 37°C without shaking to allow cell adhesion. After this incubation time, non-adhered cells were washed twice with 200 μL sterile phosphate-buffered saline (PBS, Sigma-Aldrich Co., St. Louis, MO, United States), 10 mM pH 7.2 ± 0.1. Subsequently, 200 μL of fresh SDB were added, and the plate was incubated for 48 h at 37°C to obtain mature biofilms. Then, 200 μL of AmB dilutions in the range from 2.5 to 400 μg mL^–1^ were added in order to evaluate antifungal activity on mature biofilms. After microplate incubation for 48 h at 37°C, the supernatant was collected for ROS, RNS and OSR quantification assays (O’Toole and Kolter, 1998; Ramage et al., 2001; Maiolo et al., 2016; Marioni et al., 2016; Marioni et al., 2017). The sessile minimum inhibitory concentration 50 (SMIC50) and 80 (SMIC80) of AmB were obtained based on readout 50 and 80% reductions of the mature biofilm after being treated with AmB, respectively (Marioni et al., 2016).

### Biofilm Formation and Quantification Assay

For biofilm biomass quantification, the microplate was gently rinsed with distilled water and air dried for 24 h. Each well was stained with 200 μL of CV solution 1% (w/v) and subsequently solubilized with 200 μL of ethanol/glacial acetone (70:30) solution. Total biofilm biomass was quantified spectrophotometrically at 595 nm. The average OD from control wells (ODc, containing only SDB at pH = 6.5 ± 0.1) was subtracted from the OD of all tested wells (Marioni et al., 2016, 2017; Peralta et al., 2017). *Candida* strains may be divided into terciles to establish tentative cut-offs to classify strains as being low, moderate, or high biofilm-forming according to the following criteria: OD ≤ ODc = no biofilm producer; ODc < OD ≤ (2 × ODc) = weak biofilm producer, (2 × ODc) < OD ≤ (4 × ODc) = moderate biofilm producer and (4 × ODc) < OD = strong biofilm producer (Marioni et al., 2016, 2017; Peralta et al., 2017). The biofilm biomass unit (BBU) is an arbitrary number that is related to the optical density of the biofilm and was arbitrarily defined as 0.1 OD_595_ equal to 1 BBU (Ramage et al., 2001; Maiolo et al., 2016).

For the antifungal susceptibility assay in biofilms, growth control corresponding to non-treated wells was included in all experiments (untreated biofilms). Antibiofilm activity was expressed as BBU vs. AmB concentration. Biofilm biomass reduction (%) at each AmB concentration was calculated with respect to untreated biofilms as follows (Nithyanand et al., 2015):

$$\%\,B\,i\,o\,f\,i\,l\,m\,b\,i\,o\,m\,a\,s\,s\,r\,e\,d\,u\,c\,t\,i\,o\,n=\frac{B\,B\,U\,u\,n\,t\,r\,e\,a\,t\,e\,d-B\,B\,U\,t\,r\,e\,a\,t\,e\,d}{B\,B\,U\,u\,n\,t\,r\,e\,a\,t\,e\,d}\times 100$$

### Persister Cells Quantification Assay

Following biofilm formation and AmB treatment, the supernatant was eliminated and 150 μL of PBS (10 mM, pH 7.2 ± 0.1) were added to each well (Sun et al., 2016). The biofilms were disrupted by sonication (40 kHz, 60 s) in order to re-suspend and recover viable sessile cells, after which, serial dilutions (1:10) were made in PBS and 100 μL of each dilution was pipetted out and spread on SDA plates, and then incubated for 24–48 h at 37°C. The fraction of viable sessile cells after AmB exposure was determined by plate counting (colony-forming units per mL, CFU mL^–1^), and the percentage (%) of surviving *C. tropicalis* cells was calculated with respect to untreated cells. The death curve was expressed as the% survival vs. AmB concentration (μg mL^–1^), and the PCs phenotype was corroborated by evaluating the MIC value in each assay (LaFleur et al., 2006). In addition, CFU mL^–1^ was used for correlation studies with BBU.

### Design of Experimental Model “Biofilm 1-Biofilm 2”

The B1-B2 model was designed to study the BBU, the architecture, the cellular stress metabolites and OSR of two different *C. tropicalis* biofilms exposed to AmB at supra MIC (200 μg mL^–1^) (Figure 1). First, B1 was obtained from a planktonic culture of *C. tropicalis*, while the second biofilm (B2) was formed from PCs that survived the AmB treatment in B1. Both the biofilm formation and AmB treatment were performed in parallel in different microplates (A, B, and D), as described below. After incubation with AmB, the supernatant of plate “A” was collected to determine extracellular reactive oxygen species (eROS), RNS and OSR, while biofilm biomass was quantified by CV staining, light microscope (LM) and CLSM.

The supernatant of plate “B” was discarded and the wells were washed with PBS 10 mM (pH 7.2 ± 0.1). Then, 200 μL of SDB were added to each well, and the plate was sonicated (40 KHz) for biofilm disruption. PCs1 from AmB 200 μg mL^–1^ treated wells were recovered and a standardized new cellular suspension of entirely viable cells (1–5 × 10^7^ cells mL^–1^) was made from this fraction, after which, a second mature biofilm (B2) was obtained after 48 h of incubation (plate “D”). B2 was also treated with AmB 200 μg mL^–1^ for 48 h at 37°C. Then, the supernatant was fractioned for oxidative metabolites and antioxidant system assays, and the microplate was stained with CV for biomass quantification (plate “E”). Furthermore, the MIC, MCF and the fraction of viable biofilm cells was determined by CFU mL^–1^ counting, and the percentage of surviving cells (PCs2) was calculated relative to the untreated condition (plates “C and F”).

### Reactive Metabolite Quantification Assays

eROS production was assayed spectrophotometrically by the reduction of nitro blue tetrazolium (NBT) to form an insoluble dark blue diformazan precipitate proportional to the amount of eROS generated. Briefly, 100 μL of supernatant were incubated with 100 μL of NBT 1 mg mL^–1^ for 30 min at 37°C and OD_54__0 nm_ was measured. Results were normalized to biofilm biomass (OD_540_/BBU) (Heydorn et al., 2000; Baronetti et al., 2011; Quinteros et al., 2020). Besides, to compare the B1 and B2, the treated condition was relativized to the untreated condition (OD_540_/BBU-treated dividing by OD_540_/BBU-untreated) for each biofilms and these relative values were compared. Intracellular reactive oxygen species (iROS) was quantified using 2′,7′ dichlorodihydrofluorescein diacetate (DCFH-DA) probe (10 μM, Sigma-Aldrich Co, St. Louis, MO, United States) by CLSM. DCFH-DA diffuses across membranes and is hydrolyzed by intracellular esterases to DCFH (polar, non-fluorescent), which is rapidly oxidized by ROS to the highly green fluorescent 2′,7′dichlorofluorescein (DCF) when excited at 488 nm (green fluorescence intensity, FI_green_). Calcofluor-White 0.05% (v/v) (Sigma-Aldrich Co., St. Louis, MO, United States), a carbohydrate-binding fluorescent dye that stains fungal cell walls blue when excited at 405 nm, was used to quantify the biofilm biomass (FI_blue_). The results were expressed as the ratio of FI_green_ and FI_blue_ in order to normalize the iROS accumulation to biofilm biomass (Peralta et al., 2018; Quinteros et al., 2020). To obtain a comparison between both biofilms (B1-B2), the results were expressed relative to the corresponding untreated condition.

The nitric oxide (NO) production was indirectly evaluated by measuring its stable degradation products, nitrate and nitrite by the Griess reaction. Supernatant (50 μL) was mixed with 200 μL of Griess reagent (sulfanilamide 1.5% in HCl 1 N and N-1-naphthyl ethylene diamidedihidro chloride 0.13% in sterile distilled water), and OD was measured at 540 nm. NaNO_2_ 1 mM was used as the standard, and a calibration curve was obtained from 1:2 dilutions (Baronetti et al., 2011; Peralta et al., 2018; Quinteros et al., 2020). RNS/BBU values from the SMIC80-treated condition were relativized to the untreated control for both biofilms (B1-B2).

### Antioxidant System Quantification Assays

Superoxide dismutase (SOD) activity was assayed spectrophotometrically in 10 μL of supernatant. The assay used is based on the ability of SOD to inhibit the NBT reduction by the superoxide anion (O_2_^⋅^−) generated through the illumination of riboflavin in the presence of methionine (electron donor). The plate was exposed to a 20 W fluorescent lamp for 90 min and OD was subsequently measured at 595 nm (Marioni et al., 2016, 2017; Arce Miranda et al., 2019). The normalized results (% SOD activation/BBU) were expressed relative to the corresponding untreated condition in order to compare the biofilms (B1-B2). The total reduced glutathione (tGSH) was quantified spectrophotometrically by an enzymatic recycling method that uses the Ellman reactive, 5,5′-dithiobis-2-nitrobenzoic acid (DTNB). The tGSH contained in 100 μL of supernatant was oxidized by 20 μL of DTNB 1.5 mg mL^–1^ (Sigma-Aldrich Co., St. Louis, MO, United States) in the presence of 20 μL glutathione reductase 6 U mL^–1^ (Sigma-Aldrich Co., St. Louis, MO, United States) and 50 μL NADPH 4 mg mL^–1^ (Sigma-Aldrich Co., St. Louis, MO, United States). The OD of the reaction product (2-nitro-5-tiobenzoic acid) was determined at 405 nm after 30 min of incubation (Baronetti et al., 2011). Normalized values (tGSH/BBU) from the SMIC80-treated condition were relativized to the untreated samples for comparison between both biofilms (B1-B2).

Total antioxidant capacity was evaluated by the ferrous reduction antioxidant power assay (FRAP) assay, which measures the reduction of the ferric salt ferric, tripyridyltriazine (Fe^3+^-TPTZ), to a blue colored ferrous complex by antioxidants under acidic condition. The supernatant (10 μL) was mixed with 300 μL of the following mixture (10:1:1): acetate buffer (300 mM), 2,4,6-tripyridyl-s-triazine (10 mM) in HCl (40 mM) and FeCl_3_.6H_2_O (Anedra Tigre, Argentina, 20 mM). A FeSO_4_ (Anedra Tigre, Argentina) calibration curve was used and OD was measured at 595 nm (Peralta et al., 2018; Arce Miranda et al., 2019). FRAP/BBU was expressed relative to the corresponding untreated condition to compare the biofilms (B1–B2).

### Effect of ROS Quencher or Scavengers on B1 and B2

The effects of tiron as a quencher for O_2_^⋅^− (200 mM), and the two scavengers mannitol (Sigma-Aldrich Co., St. Louis, MO, United States, 100 mM) for hydroxyl radical (HO⋅) and ascorbic acid (Anedra Tigre, Argentina, 100 mM) for total ROS were assayed in both mature biofilms. Biofilm formation and antifungal activity assays were performed as mentioned above. The quencher or scavengers were added to the biofilms (B1 and B2) at the same time as AmB, and after 48 h incubation at 37°C the supernatant was removed in order to measure BBU, ROS, RNS, and OSR (Marioni et al., 2016). The following conditions were assayed: Untreated (biofilm control), Basal (biofilm formed in the presence of the quencher), AmB (mature biofilm incubated with AmB) and AmB plus quencher (mature biofilm incubated with AmB plus quencher at the same time). The reactive metabolites (ROS, RNS) and the antioxidant system (SOD, tGSH, FRAP) were normalized to the corresponding basal BBU values by division. Besides, in order to achieve an optimal expression of the results, AmB and AmB plus quencher conditions were relativized to the basal condition by division.

### Microscopic Analysis

*C. tropicalis* biofilms were developed over small glass covers (12 mm Ø, MenzelDeckgläser, Braunschweig, Germany) placed in a 24-well microtiter plate (Greiner Bio-One, Frickenhausen, Germany) treated with fetal bovine serum 50% (v/v). Following biofilm formation and AmB exposure (2.5 and 200 μg mL^–1^), the supernatant was eliminated and the discs were rinsed with sterile PBS 10 mM (pH 7.2 ± 0.1). After 48 h of incubation at 37°C, samples were washed with sterile PBS and dried at room temperature (Peralta et al., 2018; Arce Miranda et al., 2019).

For LM, samples were stained with CV 1% (p/v) for 5 min and subsequently rinsed with PBS (Quinteros et al., 2020). Images from 10 randomly selected positions using an inverted LM (Ax overt 40 C, Carl Zeiss MicroImaging GmbH, Göttingen, Germany), and analyzed independently by two investigators (MA da S and MGP). For studies by CLSM, biofilms were labeled with Calcofluor-White dye 0.05% (v/v) and incubated for 5 min at room temperature (Peralta et al., 2018; Arce Miranda et al., 2019; Quinteros et al., 2020). Then, the samples were analyzed with a FluoviewFV1000 spectral inverted CLSM (CLSM, Olympus Latin America, Miami, FL, United States). Discs were visualized with a 60x immersion lens (UPlanSApo 100X/1.40 UIS2 Olympus immersion), and optical sections were acquired at 0.5 μm intervals for the total thickness of the biofilms. A quantitative analysis of the *C. tropicalis* biofilm structure (LM and CLSM) was carried out with Fiji-ImageJ and COMSTAT image-analysis software by measuring the following parameters: bio-volume (μm^3^ μm^–2^), average micro-colony volume (μm^3^), surface to bio-volume ratio (μm^2^ μm^–3^), roughness coefficient (Ra^∗^), average diffusion distance (μm) and surface coverage (%) (Heydorn et al., 2000; Vorregaard, 2008; Baronetti et al., 2011; Peralta et al., 2018; Quinteros et al., 2020).

### Statistical Analysis

All experiments were performed in triplicate, for three independent experiments, and the averages and the numerical data are presented as means ± standard deviation. The relationship between the CV and CFU mL^–1^ assay values was calculated using the Pearson product correlation. Data were analyzed by using ANOVA followed by the Student-Newman-Keuls test for multiple comparisons. ^∗^*p* < 0.01 was considered significant for comparisons with untreated biofilms. #*p* < 0.01 was considered significant for comparisons between B1 and B2. ^&^ denotes statistical significance at *p* < 0.01 for comparisons between basal and AmB plus quencher or scavengers conditions. A graphical statistical analysis was performed using GraphPad Prism 6.0 (GraphPad Software, San Diego, CA, United States) and Microsoft Excel (Microsoft Corp., Redmond, WA, United States).

## Results

### Antifungal Activity in Planktonic and Sessile Yeast Cells

A critical issue in studying PCs is the precise identification and isolation of this population, with dose-dependent killing having been shown to be an effective and straightforward method to detect and isolate PCs (LaFleur et al., 2006; Al-Dhaheri and Douglas, 2008; Sun et al., 2016). First of all, to study the effects of AmB on PC formation, the MIC and the MFC in *C. tropicalis* planktonic fungal cells were determined. The MIC and MFC achieved the fungicidal endpoint with 0.25 μg mL^–1^ in oth *C. tropicalis* strains (NCPF 3111 strain, N° 1 and clinical strain, N° 2), which are within the normal range of susceptible strains (Pfaller and Diekema, 2012; Arastehfar et al., 2020).

*C. tropicalis* has been reported as being a potent biofilm producer, and this was investigated using 96-well plates in static biofilm models in triplicate. CV was used to stain the adherent sessile cells, thereby quantifying the microbial biomass. The mature 48 h biofilms were rinsed with fresh growth medium and challenged with serial dilutions of AmB (2.5–400 μg mL^–1^) for another 48 h, at 37°C.

**FIGURE 2:**
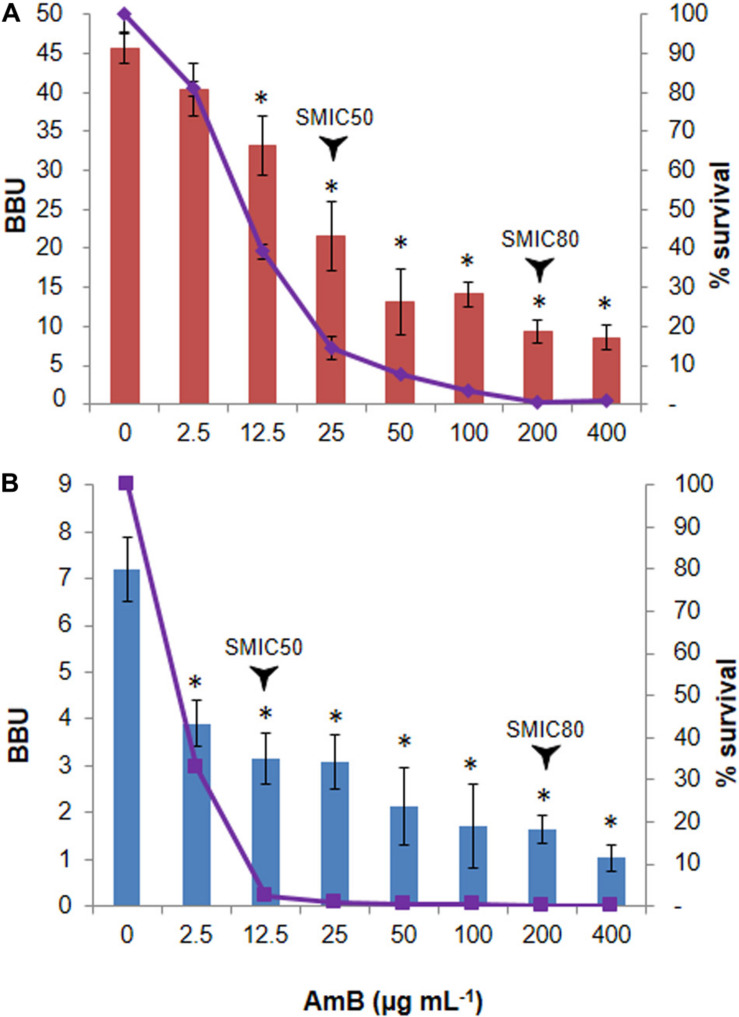
Biphasic killing pattern in response to Amphotericin B (AmB) treatment on *Candida tropicalis* biofilms. The 48 h-old biofilms of the **(A)** reference NCPF 3111-N° 1-, and **(B)** clinical-N° 2- *C. tropicalis* strains were treated with 2.5–400 μg mL^–1^ AmB. The biofilm biomass units (BBU-bar) quantified by crystal violet (CV) staining, and the fraction of surviving cells (% survival- line) calculated by viable colony-forming units, were plotted as a function of AmB concentration. Error bars represent the standard deviations of the means of three independent experiments performed in triplicate. *p < 0.01 was considered significant for comparisons with untreated biofilms.

In relation to their capacity for biofilm formation, the strain N° 1 showed to be a strong biofilm producer, whereas the N° 2 was classified as a weak biofilm producer according to the scale described in section “Materials and Methods.” Regarding the antifungal susceptibility, the SMIC50 values found were 25 μg mL^–1^ for N° 1 (Figure 2A) and 12.5 μg mL^–1^ for N° 2 strains (Figure 2B), with the SMIC80 values being 200 μg mL^–1^ for both strains.

### Quantification and Analysis of Persister Cells Populations

To quantify the fraction of AmB-tolerant PCs in the *C. tropicalis* biofilms, we incubated 48 h old biofilms with AmB for another 48 h at 37°C. The percentage of surviving sessile cells was then determined by counting the CFU mL^–1^. A biphasic killing pattern was obtained and revealed a small fraction of cells, corresponding to 0.39% for N°1 and 0.12% for N°2 strains, which survived high AmB concentrations, indicative of PC presence in the fungal biofilm (Figures 2A,B). Initially, the bulk of the susceptible yeast population was rapidly killed. As a result, the population was enriched in surviving PCs and the killing curve reached a plateau, which typically depicts the killing rate of PCs. The PC phenotype was confirmed by evaluating the MIC in each assay, which remained unchanged. The CV staining results showed a correlation with the CFU mL^–1^ assay (see Supplementary Figure 1).

**FIGURE 3:**
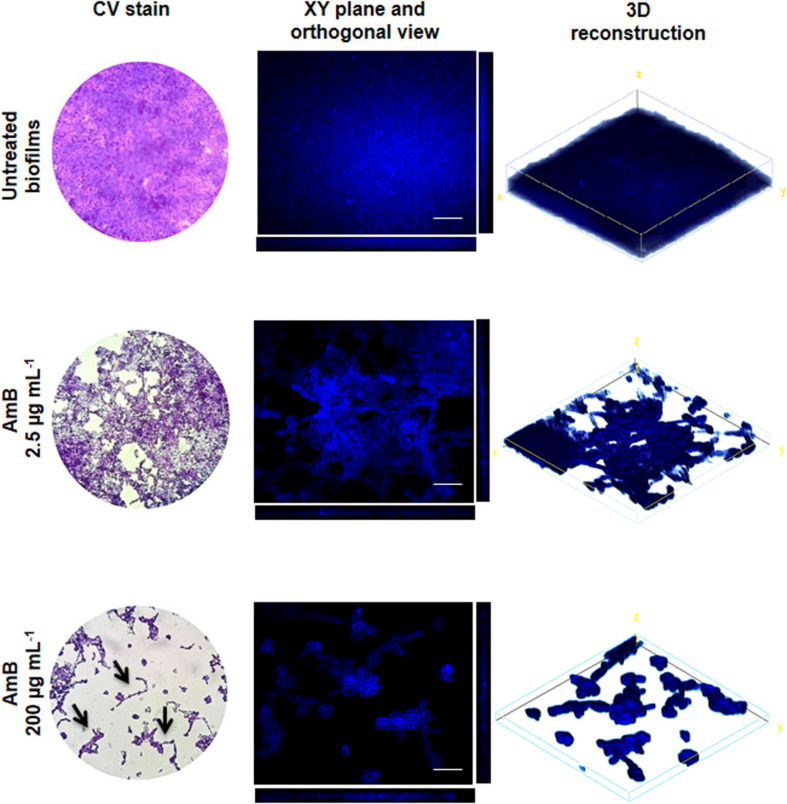
Analysis of *Candida tropicalis* NCPF 3111 biofilms by light microscope (LM) and confocal laser scanning microscopy (CLSM) images. The images correspond to untreated and treated with Amphotericin B (AmB) 2.5 and 200 μg mL^–1^ biofilms. For LM, samples were stained by crystal violet (CV) and observed at 400× magnification. CLSM images show XY (top) and XZ (bottom) planes and the 3D image reconstruction of biofilms stained with Calcofluor-White and observed at 600× magnification. Scale bar**:** 10 μm.

A LM analysis of CV-stained biofilms of *C. tropicalis* NCPF 3111, strain N° 1, was performed (Figure 3). This strain was chosen for future assays as it had been observed to be a strong biofilm producer according to the scale described in “Materials and Methods” section. The percentage area covered by the biofilms was calculated by using FIJI-ImageJ. The images produced of the untreated biofilms showed a complete covering the entire field (100% covered surface), with diffuse zones characteristic of extracellular matrix (ECM) presence, but without a clear delimitation of the sessile cells. The AmB 2.5 μg mL^–1^ treatment produced a biomass reduction, revealing a covered surface of 80–85% compared to untreated biofilms (^∗^*p* < 0.01). In addition, continuous macro-colonies were observed with a few empty spaces between colonies, formed mainly by yeast cells. Finally, at an AmB concentration of 200 μg mL^–1^ (SMIC80), the biomass decreased markedly to occupy less than 10% of the covered area, showing a loss of the cellular aggrupation and the ECM characteristic of biofilms (^∗^*p* < 0.01). A prevalence of micro-colonies was observed with very few macro-colonies and the presence of mostly yeast cells, with the appearance of some pseudo- and true hyphae (arrows—Figure 3). The biofilm architecture was further characterized by CLSM, which was used to obtain information about the topography, biofilm organization and the amount of ECM, as well as sessile cell morphological details and the spatial localization and organization of the sessile cells inside the biofilms. Figure 3 shows the CLSM images for the XY (top) and XZ (bottom) planes, and also three-dimensional reconstruction images of the topographic surfaces and architecture. These demonstrate the different decreases observed in the mature biofilm thickness when treated with different concentrations of AmB (2.5 and 200 μg mL^–1^).

### Experimental Model “Biofilm 1-Biofilm 2” for Redox Imbalance Assays

**FIGURE 4:**
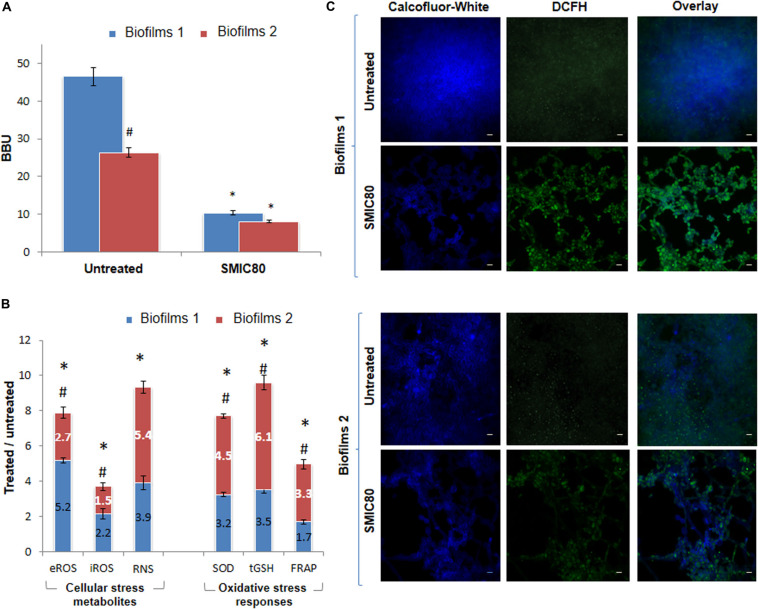
Quantification of *Candida tropicalis* NCPF 3111 biofilms after treatment with sessile minimum inhibitory concentration 80 (SMIC80) of Amphotericin B (AmB). **(A)** Biofilm biomass units (BBU) of biofilms 1 (B1) formed from planktonic culture cells and exposed to AmB (200 μg mL^–1^) and biofilms 2 (B2) formed from persister cells (PCs1) that survived the B1 treatment, and were exposed to a second AmB treatment (200 μg mL^–1^). **(B)** Cellular stress metabolites: extracellular and intracellular reactive oxygen species (eROS and iROS) and reactive nitrogen species (RNS) with respect to untreated biofilms. Oxidative stress responses (OSR): superoxide dismutase (SOD), total reduced glutathione (tGSH) and total antioxidant system determined by ferrous reduction antioxidant power (FRAP) assay with respect to untreated condition for B1 and B2 of *C. tropicalis*. **(C)** Confocal scanning laser microscopy (CLSM) images of untreated B1 and B2 and after treatment with AmB at SMIC80. Blue channel shows Calcofluor-White in sessile cell walls, and green channel shows the oxidation of the 2′,7′dichlorodihydrofluorescein diacetate probe (DCFH-DA) as an indicator of ROS production inside the biofilms. iROS was quantified as the 2′,7′dichlorofluorescein (DCF) fluorescence by using NIH-Image J. CLSM images showing two-color (blue and green) merge are shown, with a magnification of 600× and scale bar of 5 μm. All experiments were performed in triplicate, for three independent experiments, and the numerical data are presented as means ± standard deviation. *Denotes statistical significance at *p* < 0.01 for differences when compared with untreated biofilms. ^#^*p* < 0.01 differences considered significant for comparisons between B1 and B2.

The experimental model “Biofilm 1-Biofilm 2 (B1– B2)” allowed us to compare biofilm formation as well as ROS and RNS production and OSR in the *C. tropicalis* biofilms formed from planktonic cells or PCs after AmB treatment. Figure 4A shows the BBU values for *C. tropicalis* NCPF 3111 (N°1) biofilms untreated and treated at SMIC80. For untreated conditions, B2 (BBU = 26.35 ± 0.51) was significantly less (47%, #*p* < 0.01) than B1 (BBU = 46.52 ± 0.65).

In addition, the AmB treatment resulted in a biofilm biomass reduction of 78% in B1 compared to the untreated biofilms (^∗^*p* < 0.01). However, this reduction was less marked in B2 (69%) (^∗^*p* < 0.01). The surviving persister population (PCs1) from B1 produced a new biofilm (B2) which gave rise to a similar small persister population (PCs2) after a second AmB SMIC80 exposure. The MIC and MFC of the PCs were corroborated in all the assays and remained unchanged (0.25 μg mL^–1^).

An analysis of six variables describing the topography and the three-dimensional architecture of the biofilms was carried out using the software COMSTAT (Table 1). The architectural parameter values of bio-volume, average micro-colony volume, surface to bio-volume ratio, Ra^∗^, average diffusion distance and surface coverage were obtained in untreated and treated (SMIC80) samples of both B1 and B2. Comparing these values with results obtained by CV staining (BBU), the B1 bio-volume was found to have decreased by 78.2% upon SMIC80 treatment with respect to the untreated biofilms (^∗^*p* < 0.01). The average micro-colony volume, surface to bio-volume ratio and surface coverage also showed reductions of 79.5, 12.7, and 63.8%, respectively (^∗^*p* < 0.01). In addition, increases were observed of 31.5% in Ra^∗^ and 219.3% in average diffusion distance compared to untreated B1 (^∗^*p* < 0.01). AmB xposure at SMIC80 also caused important structural changes in the B2 architecture. The biofilm bio-volume and average micro-colony volume decreased by 61.5 and 50.1% (^∗^*p* < 0.01) in comparison with untreated B2, respectively. The surface coverage was also reduced (55.5%, ^∗^*p* < 0.01) whereas the surface to bio-volume ratio, Ra^∗^ and average diffusion distance showed increases of 34.2, 30.6, and 246.6% (^∗^*p* < 0.01) compared to the untreated biofilms. The analysis of these quantitative parameters demonstrated that AmB treatment at SMIC80 caused not only bio-volume reduction, but also significant structural changes in the biofilm topography and architecture.

**TABLE 1:** COMSTAT analysis of architectural parameters of *Candida tropicalis* NCPF 3111 biofilms.

	Biofilms 1		Biofilms 2	
	Untreated	AmB SMIC80	Untreated	AmB SMIC80
Bio-volume (μm^3^ μm^–2^)	2.69 ± 0.23	0.59 ± 0.05*	1.43 ± 0.03^#^	0.55 ± 0.02*
Average micro-colony volume (μm^3^)	3.63 ± 0.28	0.75 ± 0.021*	1.30 ± 0.06^#^	0.65 ± 0.02*^#^
Surface to bio-volume ratio (μm^2^ μm^–3^)	7.21 ± 0.27	6.30 ± 0.29*	5.62 ± 0.09^#^	7.55 ± 0.18*^#^
Roughness coefficient (Ra*)	1.00 ± 0.01	1.32 ± 0.02*	1.00 ± 0.01	1.31 ± 0.02*
Average diffusion distance (μm)	0.07 ± 0.00	0.21 ± 0.01*	0.05 ± 0.00^#^	0.18 ± 0.01*
Surface coverage (%)	96.57 ± 5.30	35.03 ± 1.70*	98.74 ± 1.63	43.89 ± 1.50*^#^

All experiments were performed in triplicate, and numerical data are presented as means ± standard deviation. *Denotes statistical significance at p < 0.01 for comparisons with untreated biofilms. ^#^Denotes statistical significance at p < 0.01 for comparisons between AmB-treated biofilms (B1 and B2).

Untreated biofilms (B1 and B2) were also compared using the software COMSTAT. In this sense, significant differences in bio-volume and average micro-colony volume were observed, which were 47 and 64% lower in B2 than B1 (#*p* < 0.01), respectively. In addition, the surface to bio-volume ratio and average diffusion distance parameters showed a 20% greater reduction in B2 compared to B1 (#*p* < 0.01). On the other hand, the comparison between AmB-treated biofilms revealed similar bio-volume values in both biofilms, with the average micro-colony volume being 10% lower in B2 (#*p* < 0.01). The surface to bio-volume ratio and surface coverage were 20% higher in B2 (#*p* < 0.01), while the Ra^∗^ and average diffusion distance did not change significantly. As a bio-volume reduction generates empty areas (holes) inside the biofilm structure, thereby increasing the exposure of the cells to the surface or interface, this may explain the increase in the surface to bio-volume ratio. Finally, hyphal growth occupied a greater surface than its yeast counterpart form and may have caused an increase in the surface coverage percentage.

In order to establish the PC effects on oxidative imbalance and biofilm reduction in response to AmB exposure, the eROS, iROS, NO, SOD, tGSH, and total antioxidant capacity of *C. tropicalis* NCPF 3111 (N°1) biofilms were examined, with the untreated and AmB SMIC80 treated conditions being compared in both B1 and B2 (see Supplementary Figure 2). The eROS measurement showed an increase of 5.2- and 2.7-fold compared to untreated levels in B1 and B2 (^∗^*p* > 0.01), respectively (Figure 4B). The iROS production was evaluated using a DCFH-DA by CLSM and Fiji-ImageJ quantitative analysis, and the DCF (FI_green_) to Calcofluor-White (FI_blue_) ratio was calculated in order to relativize to the biofilm biomass. AmB SMIC80 exposure increased iROS compared to untreated values by 2.2- and 1.5-fold in B1 and B2 (^∗^*p* > 0.01), respectively. It is interesting to note that iROS accumulation was lower than eROS in all cases, and also that the magnitude of both iROS and eROS increments induced by the antifungal treatment was lower in B2 than B1 (#*p* > 0.01) (Figure 4B). The CLSM images display the co-localization, which is observed in turquoise color as the merging of two different additive colors (Figure 4C). Since the RNS have been shown to promote cell death and biofilm dispersal, NO was measured in order to investigate RNS production in B1 and B2. Figure 4B shows that the NO generation was 3.9-fold (^∗^*p* < 0.01) and 5.4-fold (^∗^*p* < 0.01) higher in AmB treated biofilms in comparison with untreated ones of B1 and B2, respectively. However, these increases were not significantly different when comparing both biofilms.

Antioxidant non-enzymatic and enzymatic defenses are protective mechanisms that detoxify oxidative stress metabolites. SOD activity, the main enzyme involved in ROS detoxification, was found to be slightly higher in B2 than in B1 (#*p* > 0.01), with the respective increases being 4.5-fold (^∗^*p* < 0.01) and 3.2-fold (^∗^*p* < 0.01) compared to untreated B2 and B1 (Figure 4B). Furthermore, when the non-enzymatic antioxidant was evaluated, tGSH was greater in the biofilm formed from the PCs (B2) than in B1 (#*p* > 0.01), showing an increase of 6.1-fold (^∗^*p* < 0.01) and 3.5-fold (^∗^*p* < 0.01) upon AmB exposure, respectively. Total antioxidant capacity was evaluated by the FRAP assay, to determine the enzymatic and non-enzymatic antioxidant components, as well as SOD and tGSH. In B2, the SMIC80 AmB treatment provoked a 3.3-fold (^∗^*p* < 0.01) increase in the FRAP level compared to untreated biofilms, while a 1.7-fold (^∗^*p* < 0.01) increase was observed for B1. Thus, the total antioxidant capacity was found to be higher in B2 than B1 (#*p* > 0.01) (Figure 4B).

**FIGURE 5:**
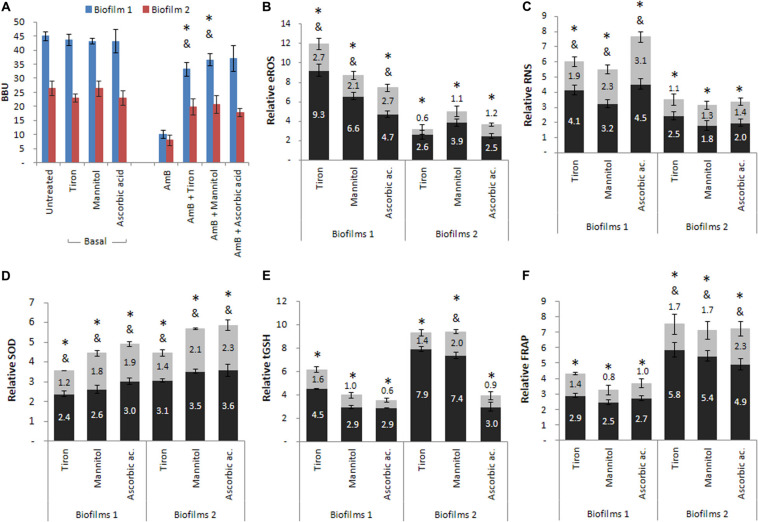
Cellular stress metabolites and oxidative stress responses (OSR) of biofilms 1 (B1) formed from planktonic culture and of biofilms 2 (B2) formed from persister cells (PCs) co-incubated in the presence of three reactive oxygen species (ROS), quencher or scavengers. Tiron, mannitol and ascorbic acid were added to **(A)** B1 and B2, and the **(B)** extracellular reactive oxygen species (eROS), **(C)** reactive nitrogen species (RNS), **(D)** superoxide dismutase (SOD), **(E)** total reduced glutathione (tGSH) and **(F)** total antioxidant system were determined by the ferrous reduction antioxidant power (FRAP) assay. Black bars correspond to AmB treated conditions relative to the basal condition and gray bars correspond to AmB plus quencher condition relative to the corresponding basal condition. All experiments were performed in triplicate, for three independent experiments, and the numerical data are presented as means ± standard deviation. *denotes statistical significance at *p* < 0.01 for comparisons between conditions basal and AmB, and & denotes statistical significance at *p* < 0.01 for comparisons between basal and AmB plus quencher or scavengers conditions.

Blocking the oxidative pathway has been very useful for evaluating the generation of oxidative stress. Here, we used tiron as a quencher for O_2_⋅−, mannitol as a scavenger for HO⋅, and ascorbic acid to scavenger the total ROS. For basal B1 and B2, the presence of the quencher or scavengers did not significantly affect biofilm formation. In addition, the biofilm reduction induced by the treatment with AmB in B1 and B2 was reverted when the quencher or scavengers were added together with AmB (Figure 5A). The BBU restitution was partial in B1 (^∗^*p* < 0.01) except for ascorbic acid, whereas it was total for B2 (see Supplementary Table 2). The production of the ROS and RNS metabolites showed similar profiles, with an increase observed after treatment with AmB 200 μg mL^–1^ in both biofilms (^∗^*p* < 0.01). Moreover, the incubation with the three scavenger compounds revealed a partial decrease in the metabolites accumulation in B1 (^&^*p* < 0.01), while in B2 they decreased until reaching the basal levels (Figures 5B,C).

For the SOD activity (Figure 5D), a greater activation was found in B1 and B2 in response to treatment with AmB 200 μg mL^–1^ (^∗^*p* < 0.01), whereas the presence of the three quenchers revealed a partial decrease in all cases without reaching basal levels (^&^*p* < 0.01). The tGSH levels (Figure 5E) were increased after treatment with AmB 200 μg mL^–1^ with respect to basal biofilms in all cases (^∗^*p* < 0.01). The incubation of AmB with the quencher or scavengers resulted in a decrease in tGSH levels compared to the previous condition (AmB), reaching values similar to the basal levels in both biofilms, except for mannitol in B2, in which the decrease was partial (^&^*p* < 0.01). Finally, Figure 5F shows the effect of these three compounds on the total antioxidant capacity (enzymatic and non-enzymatic) of the system evaluated by FRAP. In the presence of the quencher or scavengers, the levels of FRAP partially decreased in B2 (^&^*p* < 0.01), while in B1, the levels observed were similar to the basal biofilms.

In summary, we observed that the addition of tiron, mannitol, and ascorbic acid reversed the effects produced by AmB in both biofilms. The percentages obtained were greater than 75% in all cases (see Supplementary Table 1), indicating that the generation of oxidative and nitrosative stress plays an important role in *C. tropicalis* biofilms. Comparing both biofilms, it should be emphasized that the reversal in B2, originated from PCs, was total in the presence of the three compounds, while in B1, formed by planktonic cells, this effect was only observed with ascorbic acid. Concerning the evaluation of enzymatic and non-enzymatic antioxidant defenses, we observed that these defenses were also less active in the presence of quencher or scavenger compounds. However, the SOD enzyme maintained a higher activation in B2, but this was not observed for tGSH, which decreased to basal levels in both biofilms. In addition, the total antioxidant capacity of samples was estimated by the FRAP, with the biofilm formed from PCs (B2) revealing a greater capacity to respond to oxidative stress than the biofilm from planktonic cells (B1) after AmB treatment.

## Discussion

A direct link between the recalcitrance of recurrent or chronic infections and PCs has been recently described (Lewis, 2008; Fauvart et al., 2011; Conlon et al., 2013; Koo et al., 2017; Defraine et al., 2018). Concerning PCs, most of the studies on PCs subpopulations in the genus *Candida* have been carried out in *C. albicans*, while other species have been far less studied. For *C. tropicalis*, in particular, conflicting results have been found. Al-Dhaheri and Douglas reported an absence of PCs in biofilms of clinical *C. tropicalis* strains treated with AmB concentrations of up to 100 μg mL^–1^ (Al-Dhaheri and Douglas, 2008). In contrast, Li et al. (2015) reported PCs in biofilms of both the reference and clinical *C. tropicalis* strains upon treatment with AmB up to 250 μg mL^–1^, in between 0.01 and 0.05% of the total cell population, similarly to that reported in *C. albicans* and *C. glabrata* biofilms. Similarly to most investigations concerned to study, in this work we have performed dose-dependent killing curves in order to evaluate the presence of CPs in biofilms of the studied *C. tropicalis* strains, as it has demonstrated to be an effective and reliable method to detect and isolate PCs (LaFleur et al., 2006; Al-Dhaheri and Douglas, 2008; Sun et al., 2016). Our results show that *C. tropicalis* mature biofilms contained a small fraction of cells tolerant to AmB concentrations of at least 200 μg mL^–1^ in both the reference (0.39%) and clinical (0.12%) strains. The characteristic biphasic killing curve showed an initial rapid decline in the slope, representing the death of regular sessile susceptible cells, which was followed by a slower killing trend indicative of the surviving PC subpopulation presence, with this killing pattern being a determining indicator of PCs in both strains. These results are similar to those reported by other authors, who also demonstrated the existence of PCs in several *Candida* spp. strains, such as LaFleur et al. (2010), who obtained PC subpopulation values of between 0.05 and 2% of the total cell population. Moreover, Truong et al. (2016) obtained a value close to 1% in *C. albicans* biofilms. Another study described the presence of PCs in *C. albicans* (0.01%), *C. parapsilosis* (0.07%), and *C. krusei* (0.001%) (Al-Dhaheri and Douglas, 2008). In all these cases, the persistent phenotype was verified by the MIC values of the cells recovered after antifungal treatment, which remained unchanged, showing that they were transient phenotypic variants instead of resistant cells. Our results also confirmed that only the PCs remained in *C. tropicalis* biofilms after two successive AmB treatments at SMIC80, and exhibited the same sensitivity (MIC and MFC) as the original strain upon regrowth.

The B1-B2 model used in this study allowed us to carry out the CLSM studies and COMSTAT quantification, and to analyze structural differences between both biofilms. This analysis showed agreement with the BBU values obtained by CV staining. The most significant difference found between both biofilms was related to their size, with B2, formed from PCs1, being substantially smaller than B1 and having 47 and 64% less biomass and thickness, respectively (#*p* < 0.01). This might be explained at least in part in view of the different oxidative (redox) status of the PC fraction that gave rise to B2. Balaban et al. (2004) demonstrated that PC reactivation may result in a more pronounced lag phase with respect to untreated planktonic cells. Likewise, it is possible that PCs recovered from B1 required a longer period of time to reach the normal levels of metabolic activity involved in biofilm formation and development. However, the smaller size of this second biofilm did not influence PC formation, since after the second treatment with AmB 200 μg mL^–1^, a new persistent population (PCs2) of a similar size was found and was this corroborated by CFU counting and the MIC values. Regarding the AmB SMIC80 treatment, one important difference between both biofilms was related to cell morphology. In B2, hyphae and pseudohyphae, in addition to budding yeasts, increased the surface coverage (%) and surface to bio-volume ratio obtained by COMSTAT analysis. In fact, the ability of *Candida* to switch between yeast, pseudohyphal and hyphal growth forms (polymorphism) is a critical virulence determinant, which plays a key role in the infection process, and can promote tissue invasion and resistance of host immune cells (Pappas et al., 2018).

Recent studies have suggested that some active cellular processes are critical for PC survival, including active drug efflux and protection against ROS (Cohen et al., 2013; Wood, 2015; Harms et al., 2016). Several reports have also indicated the possibility of enhancing the fungicidal activity of ROS-inducing antimicrobials in planktonic cultures by disrupting the OSR. For example, an increased activity of antifungals targeting the oxidative and osmotic stress response systems against several yeasts and filamentous fungi was observed using benzaldehydes and benzoic acids (Kim et al., 2008). Wu et al. (2012) noted the ability of oxidative stress induced by paraquat, an oxidative stress inducer, to produce an important increase in the number of PCs in *E. coli* cultures treated with a fluoroquinolone, suggesting that stress responses may also contribute to PC formation. Using the *in vitro* B1-B2 model, we studied the ROS, RNS and OSR profiles of *C. tropicalis* biofilms from planktonic cells (B1), and compared these with a second biofilm derived from the PC fraction obtained upon AmB SMIC80 exposure (B2). The AmB treatment induced ROS and RNS generation and produced a redox imbalance in sessile cells in both biofilms. We observed a similar increase in the eROS:iROS ratio in both biofilms, demonstrating the induction of generalized stress, with an important eROS accumulation in the ECM.

Several proteins involved in oxidative stress defenses, including SOD and glutathione reductase, are up-regulated in biofilms, which may contribute to the higher resistance to ROS-inducing antifungals such as AmB (Cohen et al., 2013). In the present study, the antioxidant response between the two biofilms was compared and it was found that the one originating from PCs presented a higher OSR than the biofilms formed from planktonic cells not previously exposed to AmB. The SOD, tGSH, and FRAP levels were observed to be greater in B2, indicating a differential induction of these parameters in the biofilm derived from PCs. Among these indicators, the greatest difference (respect to B1) was observed at the level of the non-enzymatic component. In agreement, a lower accumulation of eROS was observed in B2, indicating that the main inactivation pathway of this metabolite occurred through the successive action of the SOD enzyme followed by the glutathione peroxidase/glutathione reductase (GPX/GR) duplex using GSH as the cofactor. The PC population may be responsible for a higher OSR in the *C. tropicalis* biofilms. However, this was not sufficient to maintain the cellular redox balance, resulting in general oxidative stress, with a concomitant biomass reduction of biofilms, as previously reported (Kim et al., 2008; Seneviratne et al., 2008; Wood, 2015). In agreement with our result, SOD was reported to be involved in *C. albicans* biofilm protection against AmB and miconazole, probably via the detoxification of antifungal-induced O_2_⋅− (Kim et al., 2012). Miconazole treatment of *C. albicans* with impaired SOD also results in a decrease of PCs (Bink et al., 2011). Bink et al. (2011) demonstrated an important role for SODs in the occurrence of miconazole-tolerant PCs by using the Cu, Zn-SOD inhibitor N,N-diethyldithiocarbamate, resulting in reduced PC levels in miconazole-treated *C. albicans* biofilms and a rise in the ROS levels. Similarly, De Brucker et al. (2011) also demonstrated the importance of SOD in the tolerance of *C. albicans* biofilms exposed to AmB, observing that inhibition of this enzyme directly affected the PC fraction obtained. The results derived from our model demonstrated that both B1 and B2 presented different redox status, and therefore, a differential capacity to respond to the oxidative and nitrosative stress generated by AmB. The oxidative stress generated was directly related to the percentage of reduction obtained for each biofilm (78 and 69% for B1 and B2, respectively), leading to a new population of PCs (PC2) from B2, which remained viable after the second AmB treatment. This has also been observed by other authors when performing successive treatments of AmB or chlorhexidine at high concentrations on *C. albicans* biofilms (LaFleur et al., 2010; Mesa-Arango et al., 2014).

The use of exogenous antioxidant quenchers or scavengers has been postulated as being very useful for evaluating the generation of oxidative stress and cell viability. Li et al. demonstrated that the addition of mannitol, which acts as an HO⋅ scavenger, reduces antifungal activity by UV-LED irradiation of *E. coli* and *C. albicans* biofilms (Li et al., 2010). Other authors have shown that tiron decreases the accumulation of intracellular O_2_^⋅^− in fibroblasts exposed to *Clostridium perfringens* toxin (Monturiol-Gross et al., 2012). Furthermore, treating *Mycobacterium* species under aerobic conditions or combined with clofazimine, a ROS inducer, decreased the surviving fraction under antibiotic treatment, while the addition of thiourea, a HO⋅ scavenger, led to a higher PC fraction (Grant et al., 2012). Aerts et al. (2007) demonstrated that ascorbic acid, a non-specific ROS scavenger, blocks the induction and consequently the antifungal effect in planktonic cultures of *C. albicans.*

In order to evaluate the role of oxidative stress in our model, treatment with AmB was carried out in the presence of a quencher or scavengers. Tiron, mannitol and ascorbic acid reduced the oxidative stress caused by AmB in *C. tropicalis* NCPF 3111 biofilms (B1 and B2), reverting the biofilm reduction induced by the treatment with AmB alone. In addition, a differential response was observed in the biofilms (B1 and B2), with the reversion of the antifungal effect being total in the biofilms derived from a persistent fraction (B2) for the three compounds used, whereas in the biofilms originating from planktonic cells (B1), this reversion was complete only with ascorbic acid, the non-specific ROS scavenger. Thus, the use of these compounds showed that oxidative and nitrosative stress generation was key in the antifungal effect of AmB on the *C. tropicalis* biofilms. In addition, some investigations have described a differential induction of certain proteins in the PC fraction of *C. albicans* biofilms, with some of these being involved in the OSR, which is key to generating antifungal tolerance in this subpopulation (Seneviratne et al., 2008; Grant et al., 2012; Guirao-Abad et al., 2017).

To our knowledge, the impact on the oxidative state of a new biofilm originating from a PC subpopulation has not been previously studied. From the results obtained in our model, these PCs can have an impact on the oxidative and nitrosative state of the new biofilms, which due to the greater antioxidant response capacity, may contribute to the resistance to a new antifungal treatment. In conclusion, this study shows for the first time that *C. tropicalis* biofilms formed from PCs exhibit an increased OSR to a second AmB exposure, and this greater capacity for ROS tolerance implies that it could benefit their prevalence and dissemination in the host. Although our current understanding of biofilm PCs is limited and the survival mechanisms of *C. tropicalis* biofilm PCs remain unclear, recognizing the importance of cellular stress imbalance will surely contribute to future investigation into this topic.

## Data Availability Statement

The raw data supporting the conclusions of this article will be made available by the authors, without undue reservation, to any qualified researcher.

## Author Contributions

MP conceptualized the project. MS and JB performed the experiments. PP and MP performed analysis and visualization of experimental results and acquired the funding. JB and MP wrote and edited the manuscript. MP supervised the project. All authors contributed to the article and approved the submitted version.

## Conflict of Interest

The authors declare that the research was conducted in the absence of any commercial or financial relationships that could be construed as a potential conflict of interest.

## Supplementary Material

Supplementary Figure 1 Percentage of surviving sessile cells (% survival) determined by colony-forming units per mL (CFU mL^–1^) count and correlating this with results obtained by crystal violet (CV) staining as biofilm biomass unit (BBU).

Supplementary Figure 2 Cellular stress metabolites and oxidative stress responses (OSR) of untreated and treated Amphotericin B (AmB) at sessile minimum inhibitory concentration 80 (SMIC80) in biofilms 1 (B1) and biofilms 2 (B2). All experiments were performed in triplicate, for three independent experiments, and the numerical data are presented as means ± standard deviation. ^∗^ denotes statistical significance at *p* < 0.01 for differences when compared with untreated biofilms. ^#^*p* < 0.01 differences considered significant for comparisons between B1 and B2. The numerical data correspond to the relative values (treated/untreated ratio) shown in Figure 4B.

Supplementary Table 1 Biofilm biomass unit (BBU) values and percentage (%) of reversion in the presence of oxidative metabolite quenchers.
