# Supplementary figures and images for: Oxidative Imbalance in Candida tropicalis Biofilms and Its Relation With Persister Cells
Source: Front Microbiol. 2021 Feb 2;11:598834. doi: 10.3389/fmicb.2020.598834 (PMC7884318; doi:10.3389/fmicb.2020.598834)

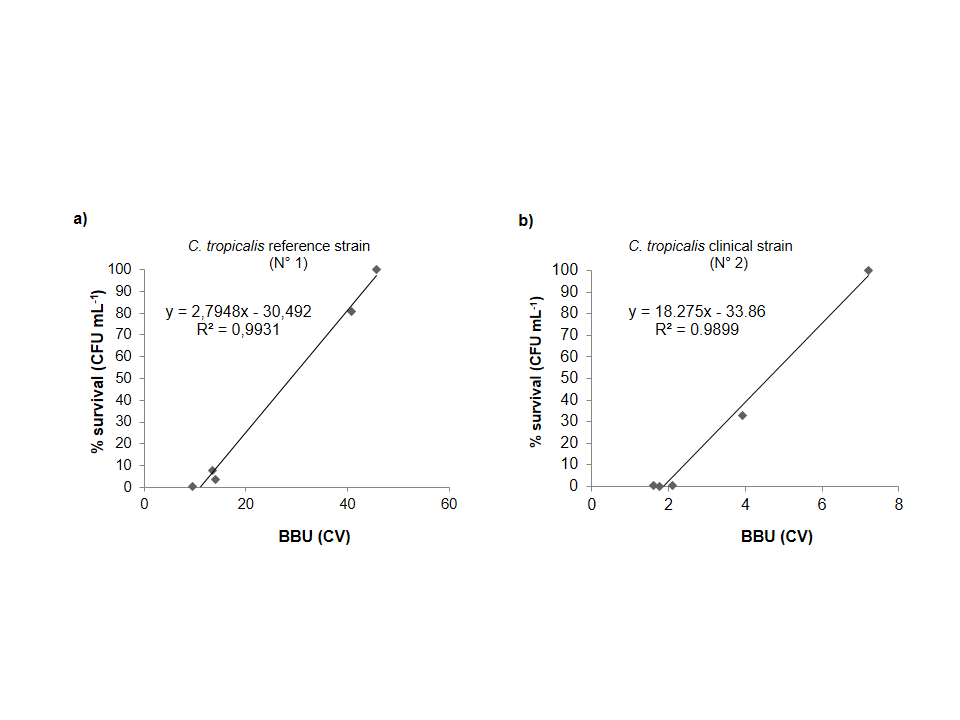

Supplement: Supplementary Figure 1 — Percentage of surviving sessile cells (% survival) determined by colony-forming units per mL (CFU mL–1) count and correlating this with results obtained by crystal violet (CV) staining as biofilm biomass unit (BBU). [file Image_1.TIF]

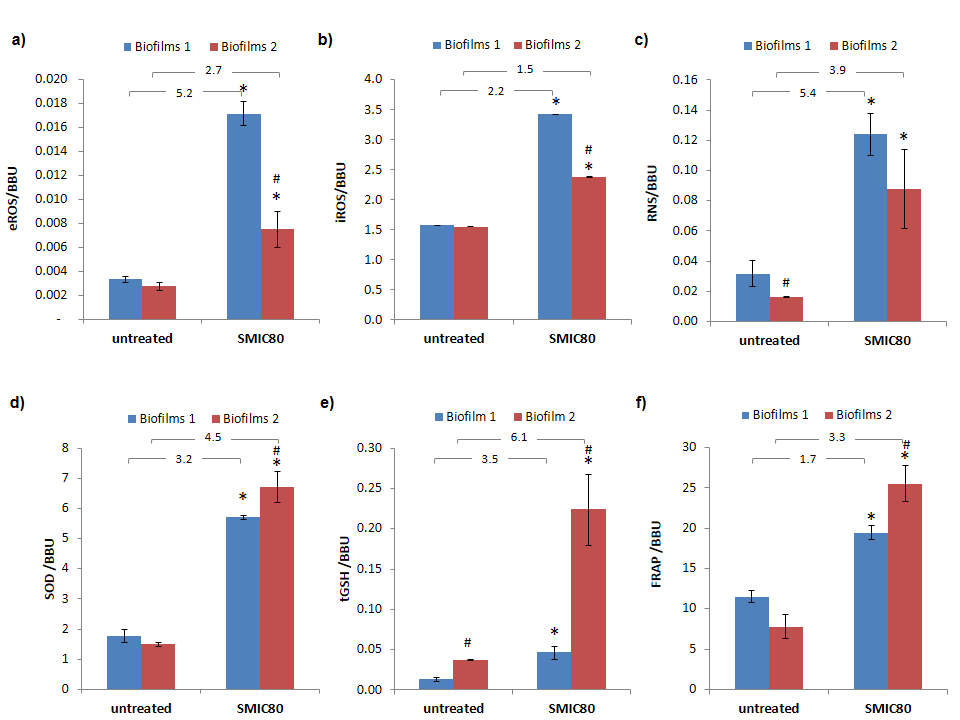

Supplement: Supplementary Figure 2 — Cellular stress metabolites and oxidative stress responses (OSR) of untreated and treated Amphotericin B (AmB) at sessile minimum inhibitory concentration 80 (SMIC80) in biofilms 1 (B1) and biofilms 2 (B2). All experiments were performed in triplicate, for three independent experiments, and the numerical data are presented as means ± standard deviation. ∗ denotes statistical significance at p < 0.01 for differences when compared with untreated biofilms. #p < 0.01 differences considered significant for comparisons between B1 and B2. The numerical data correspond to the relative values (treated/untreated ratio) shown in Figure 4B. [file Image_2.TIF]
