# Supplementary material for: Oxidative Imbalance in Candida tropicalis Biofilms and Its Relation With Persister Cells
Source: Front Microbiol. 2021 Feb 2;11:598834. doi: 10.3389/fmicb.2020.598834 (PMC7884318; doi:10.3389/fmicb.2020.598834)
Supplement: Supplementary Table 1 — Biofilm biomass unit (BBU) values and percentage (%) of reversion in the presence of oxidative metabolite quenchers. [file Table_1.DOCX]

**Supporting information Table 1: Biofilm biomass unit (BBU) values and percentage (%) of reversion in the presence of oxidative metabolite quenchers**

| **Conditions** | | **BBU (untreated + quencher)** | **BBU (AmB + quencher)** | **Reversion**  **(%)^a^** |
| --- | --- | --- | --- | --- |
| **Tiron** | B1 | 43.53 ± 1.97 | 33.16 ± 2.46*^&^ | 76 ± 12 |
|  | B2 | 22.92 ± 1.47 | 19.76 ± 2.93^&^ | 86 ± 21 |
| **Mannitol** | B1 | 43.12 ± 0.96 | 36.57 ± 2.25*^&^ | 85 ± 8 |
|  | B2 | 26.31 ± 2.70 | 20.65 ± 3.19^&^ | 78 ± 26 |
| **Ascorbic acid** | B1 | 43.14 ± 4.17 | 36.93 ± 4.61*^&^ | 86 ± 22 |
|  | B2 | 23.08 ± 2.60 | 17.74 ± 1.50^&^ | 77 ± 20 |

All experiments were performed in triplicate, and numerical data are presented as means ± standard deviation. ^a^ values (%) calculated respect to AmB treated conditions.

*denotes statistical significance at p < 0.01 for comparisons between treated and non-treated biofilms.

^&^denotes statistical significance at p < 0.01 for comparisons between treated with AmB and AmB plus quencher.
